# *All-Trans* Retinoic Acid Promotes TGF-β-Induced Tregs via Histone Modification but Not DNA Demethylation on Foxp3 Gene Locus

**DOI:** 10.1371/journal.pone.0024590

**Published:** 2011-09-13

**Authors:** Ling Lu, Jilin Ma, Zhiyuan Li, Qin Lan, Maogen Chen, Ya Liu, Zanxian Xia, Julie Wang, Yuanping Han, Wei Shi, Valerie Quesniaux, Bernhard Ryffel, David Brand, Bin Li, Zhongmin Liu, Song Guo Zheng

**Affiliations:** 1 Division of Rheumatology, Department of Medicine, Saban Research Institute, Children's Hospital Los Angeles, Keck School of Medicine, University of Southern California, Los Angeles, California, United States of America; 2 Department of Surgery, Saban Research Institute, Children's Hospital Los Angeles, Keck School of Medicine, University of Southern California, Los Angeles, California, United States of America; 3 Developmental Biology and Regenerative Medicine Program, Saban Research Institute, Children's Hospital Los Angeles, Keck School of Medicine, University of Southern California, Los Angeles, California, United States of America; 4 Key Laboratory of Living Donor Liver Transplantation, Nanjing, People's Republic of China; 5 Division of Rheumatology, Immunology and Nephrology, Zhejiang Traditional Chinese Medicine and Western Medicine Hospital, Hangzhou, People's Republic of China; 6 Immune Tolerance Center, Shanghai East Hospital, Tongji University, Shanghai, People's Republic of China; 7 UMR6218, Molecular Immunology, University and Centre National de la Recherche Scientifique, Orleans, France; 8 Research Service, Veterans Affairs Medical Center, Memphis, Tennessee, United States of America; 9 Unit of Molecular Immunology, Institute Pasteur of Shanghai, Chinese Academy of Science, Shanghai, People's Republic of China; Universidade de Sao Paulo, Brazil

## Abstract

**Background:**

It has been documented all-trans retinoic acid (atRA) promotes the development of TGF-β-induced CD4^+^Foxp3^+^ regulatory T cells (iTreg) that play a vital role in the prevention of autoimmune responses, however, molecular mechanisms involved remain elusive. Our objective, therefore, was to determine how atRA promotes the differentiation of iTregs.

**Methodology/Principal Findings:**

Addition of atRA to naïve CD4^+^CD25^−^ cells stimulated with anti-CD3/CD28 antibodies in the presence of TGF-β not only increased Foxp3^+^ iTreg differentiation, but maintained Foxp3 expression through apoptosis inhibition. atRA/TGF-β-treated CD4^+^ cells developed complete anergy and displayed increased suppressive activity. Infusion of atRA/TGF-β-treated CD4^+^ cells resulted in the greater effects on suppressing symptoms and protecting the survival of chronic GVHD mice with typical lupus-like syndromes than did CD4^+^ cells treated with TGF-β alone. atRA did not significantly affect the phosphorylation levels of Smad2/3 and still promoted iTreg differentiation in CD4^+^ cells isolated from *Smad3* KO and *Smad2* conditional KO mice. Conversely, atRA markedly increased ERK1/2 activation, and blockade of ERK1/2 signaling completely abolished the enhanced effects of atRA on Foxp3 expression. Moreover, atRA significantly increased histone methylation and acetylation within the promoter and conserved non-coding DNA sequence (CNS) elements at the Foxp3 gene locus and the recruitment of phosphor-RNA polymerase II, while DNA methylation in the CNS3 was not significantly altered.

**Conclusions/Significance:**

We have identified the cellular and molecular mechanism(s) by which atRA promotes the development and maintenance of iTregs. These results will help to enhance the quantity and quality of development of iTregs and may provide novel insights into clinical cell therapy for patients with autoimmune diseases and those needing organ transplantation.

## Introduction

All-trans-retinoic acid (atRA), a Vitamin A derivative, has profound effects on embryonal morphogenesis, vision, reproduction, cell differentiation, growth, and immune homeostasis [1]. Deficiency of vitamin A leads to exacerbation of experimental colitis [2]. In the immune system, atRA plays important roles in regulating the functions of many different cell types [3]. Vitamin A and its derivatives are capable of ameliorating several models of autoimmunity, including inflammatory bowel disease, rheumatoid arthritis, type I diabetes, and experimental encephalomyelitis [4]–[5]. In addition to the inhibitory effect of atRA on T effector cell differentiation and function, atRA has also been shown to be capable of promoting murine CD4^+^Foxp3^+^ Tregs induced by TGF-β from conventional CD4^+^Foxp3^−^ cells, either directly by enhancing TGF-β-driven Smad3 signaling in naive cells and/or indirectly by relieving the production of pro-inflammatory cytokines from murine memory effector cells [6]–[8]. Such approaches show great promise as these T cells have been shown effective in combating several immune-mediated disorders [9].

CD4^+^CD25^+^ T regulatory (Treg) cells play a critical role in establishing and maintaining self-tolerance. Therefore, enhancing the number and/or function of Tregs represents a potential treatment for patients with autoimmune disorders or those who undergo transplant rejection. atRA can strongly increase TGF-β-induced Foxp3 expression and Treg conversion *in vitro* [6]. Under these conditions, atRA may enhance TGF-β signaling by increasing the expression and phosphorylation of Smad3. On the other hand, it has also been reported that expression of RAR can be increased through TGF-β signaling [10]. Therefore, atRA and TGF-β may cooperatively augment their mutual signaling to further enhance Foxp3 expression. However, the exact roles of atRA in these signaling pathways are less well understood.

We recently reported that while the Smad pathway plays a less important role in the differentiation of Foxp3^+^ iTregs induced by TGF-β, ERK and JNK kinases which mainly use non-Smad pathways, may play a more significant role in this process [11]. Herein, we further demonstrate that adding atRA to cultures containing TGF-β not only increases Foxp3 expression and maintenance, but also enhances the suppressive activities of these Tregs *in vitro* and *in vivo*. Studies of the underlying mechanism responsible for these observations indicate that atRA upregulates ERK rather than Smad2/3 activation of the TGF-β down-stream signaling pathway. Additionally, Foxp3 induced by a combination of atRA and TGF-β displayed increased Foxp3 binding ability on chromatin compared to that induced by TGF-β alone. We further found that atRA enhances histone methylation and acetylation in Foxp3 promoter and its conserved non-coding DNA sequence elements (CNS2), rather than DNA CpG demethylation of CNS3 in the Foxp3 locus. Thus, atRA improves both the quantity and quality of Foxp3^+^ iTregs, findings which will be important in the development of superior cell therapies to treat autoimmune diseases and prevent organ transplantation rejection.

## Results

### atRA directly up-regulates Foxp3 and sustains its expression by CD4^+^ cells treated with TGF-β

In agreement with previous reports [6], addition of atRA to cultures containing TGF-β significantly enhanced the proportions of CD4^+^CD25^+^Foxp3^+^ cells induced from naive CD4^+^CD25^−^Foxp3^−^ (or GFP^−^ cells using WT or Foxp3 GFP knock-in mice). This effect may reflect either direct Foxp3^+^ cell induction or a secondary effect through suppression of CD4^+^Foxp3^−^ cell expansion [8]. In either case, total Foxp3 protein levels and Foxp3^+^ cell numbers increased significantly in CD4^+^ cells treated with the combination of atRA and TGF-β than those treated with TGF-β alone, indicating that atRA has a direct effect on the enhancement of iTreg differentiation, although it may also simultaneously suppress CD4^+^Foxp3^−^ cell expansion (**Figure S1A–D**).

We also examined other phenotypic features related to Treg differentiation. As reported by others [12], addition of atRA to TGF-β markedly increased the expression of CD103, CCR-9, α_4_β_7_ by CD4^+^ cells (**Figure S1E**), we also observed that addition of atRA significantly decreased IL-2 production, increased membrane bound TGF-β expression and slightly increased IL-10 production (**Figure S1E**).

**Figure 1:**
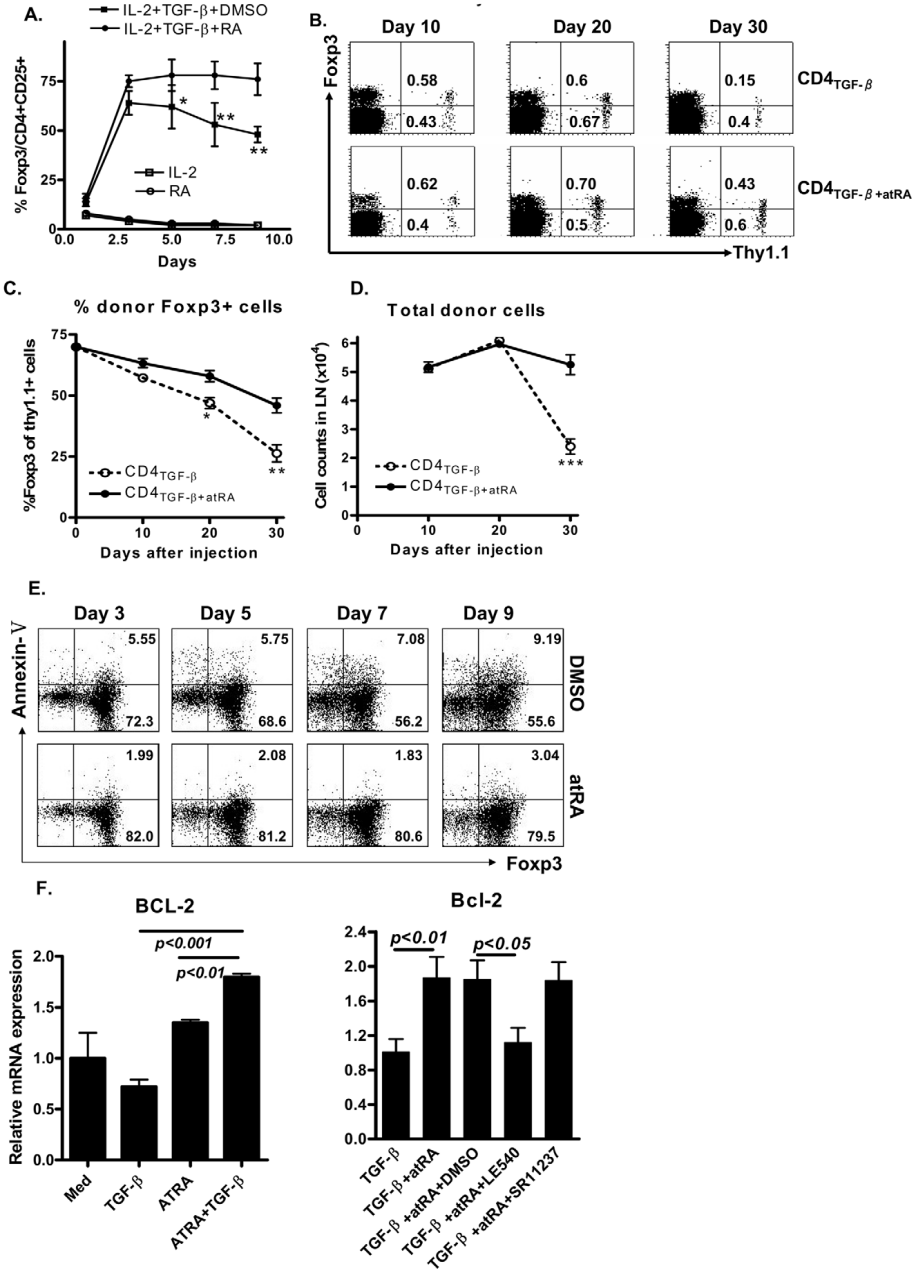
atRA sustains Foxp3 expression *via* apoptosis inhibition. (A) Splenic naïve CD4^+^ cells isolated from Foxp3^gfp^ mice were stimulated as described in the Methods section. Percentages of GFP^+^ among CD4^+^ cells were determined by FACS at the time points indicated. Values were Mean ± SEM of three separate experiments. (B). Splenic naïve CD4^+^ cells isolated from Thy1.2 C57BL/6 mice were stimulated with anti-CD3/CD28 coated beads (1∶5 ratio) with IL-2 and TGF-β ± atRA for 4 days and injected to Thy1.2 syngeneic mice. 10, 20, and 30 days later, mice were sacrificed and Foxp3 expression in the donor cells (Thy1.1^+^) in spleen, blood and lymph nodes was determined by FACS. Representative Foxp3 expression data from LN cells of one mouse per group (n = 4). The experiment was repeated with similar results. The percentages of Foxp3^+^ cells in specific donor cell populations (C) and total donor cells (D) in days post injection as indicated are shown. Values were Mean ± SEM of 8 mice in each time point. (D) The experiment was conducted as panel A and the expression of Annexin-V^+^ among GFP^+^ cells were determined by FACS at the time points indicated. Data is representative of three separate experiments. (F) Total RNA was isolated from different cell groups in panel A and expression of Bcl-2 mRNA was quantitatively determined by qPCR. Data are Mean ± SEM of triplicate wells and representative of three different experiments with similar results. *, p≤0.05; **, p≤0.01; ***, p≤0.001, CD4_TGF-β_ in comparison to CD4_atRA+TGF-β_.

In addition to a direct effect of atRA on Foxp3^+^ cell differentiation, atRA also maintains Foxp3 expression in TGF-β-primed CD4^+^CD25^+^ cells. As shown in **Figure 1A**, Foxp3 expression in CD4^+^CD25^+^ cells treated with TGF-β appeared on day 1 and peaked on day 3–5. Foxp3 expression gradually declined after 6 days in the culture and remained at lower levels on day 10. Of note, the addition of atRA not only increased, but sustained Foxp3 expression by TGF-β-primed CD4^+^ cells. We conducted further *in vivo* experiments to determine whether both Treg cell subsets have different fates after cell transfer. iTregs were generated as described above from C57BL/6 Thy1.1 mice and adoptively transferred into syngeneic C57BL/6 Thy1.2 mice. Spleen, blood and lymph node (LN) cells were stained for Foxp3 and Thy1.1 on day 10, 20 and 30 after cell transfer. Thy1.1 expression is used to distinguish the donor cells from recipient cells. While total donor CD4_TGF-β_ cells dramatically declined on day 30, the Foxp3^+^ cell subset from these cells significantly decreased on day 20 and even more on day 30 after cell transfer in LNs (**Figure 1B-D**), blood and spleen (not shown). In sharp contrast, total donor CD4_TGF-β+atRA_ cell numbers are sustained during 10–30 days after cell transfer. Although the percentages of Foxp3^+^ population among CD4_TGF-β+atRA_ cells was slightly lower in day 20-30 than in day 0, the Foxp3^+^ population was still significantly higher in donor CD4_TGF-β+atRA_ cells than in CD4_TGF-β_ cells in LNs (**Figure 1B–C**). This phenonemon was similarly observed in peripheral blood and spleen, excluding the possibility that the re-distribution of donor cells affects the frequency of Treg cells in the different donor cell populations post injection. We further observed that the addition of atRA to TGF-β-treated culture significantly decreases the proportion of Annexin-V^+^GFP^+^ (apoptotic Foxp3^+^) cells (**Figure 1E**) and up-regulates the expression of Bcl-2 (an anti-apoptotic gene) (**Figure 1F, left panel**), suggesting that atRA maintains Foxp3 expression through its effect on protecting these cells from apoptosis. Although atRA induces cancer cell apoptosis and contributes to cancer treatment [13], it actually suppresses apoptosis in non-tumor human cells including lymphocytes, eosinophils and neuronal cells [14]. We further demonstrated that atRA/RAR rather than atRA/RXR signal pathway is crucial for the upregulation of Bcl-2 expression since additon of LE540 (an RAR antagonist) rather than of SR11237 (an RXR antagonist) abolished the effect of atRA on Bcl-2 upregualtion (**Figure 1F**, right panel). This is in line with a previous report concluding that the atRA/RAR but not atRA/RXR signal pathway is important for Foxp3 induction [15].

### CD4^+^ cells primed with atRA and TGF-β displayed increased suppressive activities *in vitro* and *in vivo*

**Figure 2:**
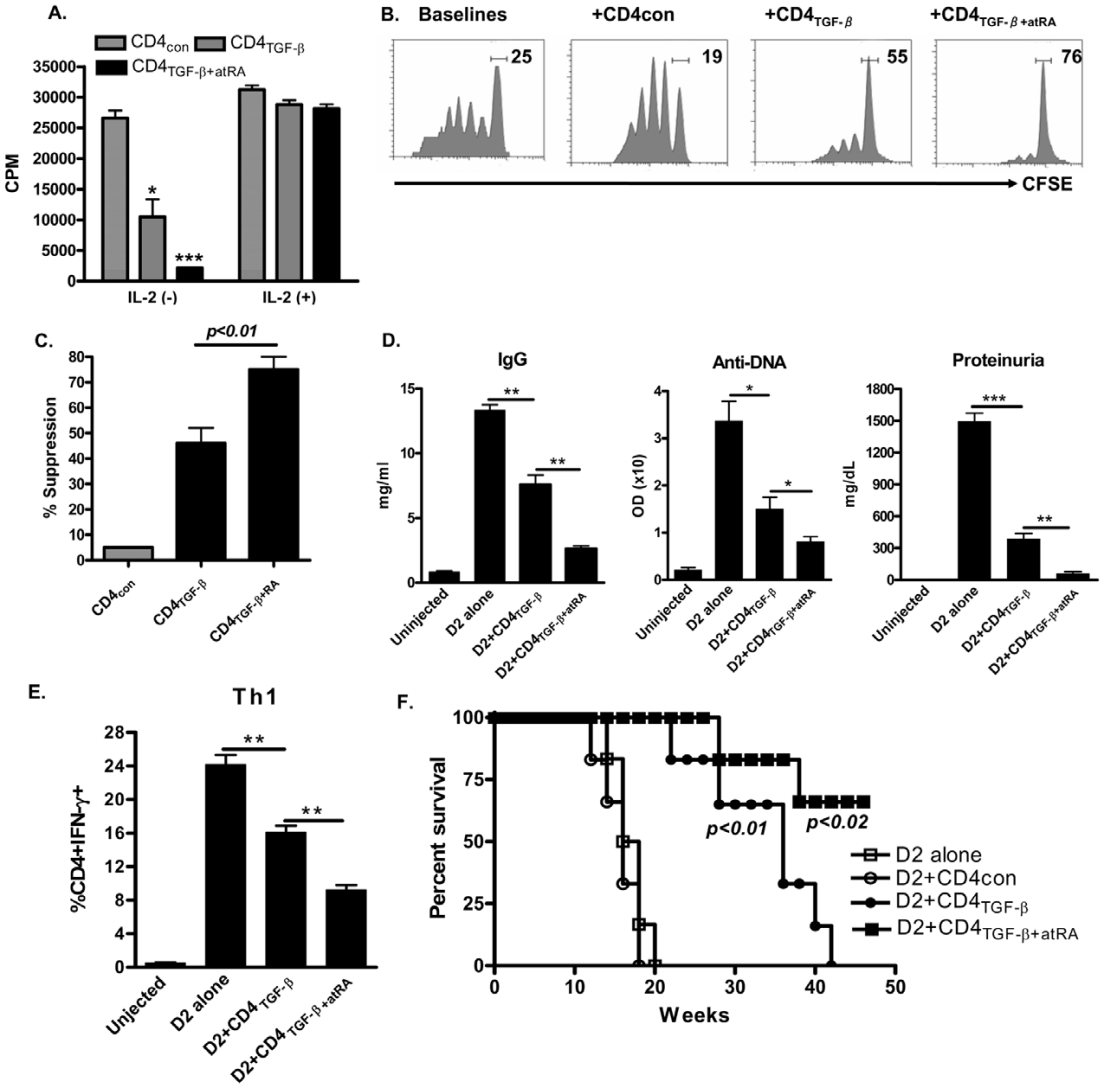
atRA enhances the suppressive activity of TGF-β-iTregs *in vitro* and *in vivo*. (A) CD4+ conditioned cells induced as in Fig. 1 were stimulated with anti-CD3 in the presence of APC for three days and their proliferation were determined by ^3^H thymidine incorporation. (B, C) CFSE-labeled effector T cells were cultured at a 1∶4 ratio with CD4^+^CD25^+^ cells isolated from medium treated CD4^+^ T cells (+CD4_con_), TGF-β-CD4^+^ cells (+CD4_TGF-β_), or atRA/TGF-β-CD4^+^ cells (+CD4_TGF-β+atRA_) or without (Baselines) for 3 days. Representative CFSE plots of the T effector cells are shown as in B, with the percentages of suppression on T effector cells quantified as in C. (D) A chronic GVHD with a lupus-like syndrome was induced in D2B6F1 mice as described previously. 80×10^6^ D2 splenocytes were injected into the tail vein (D2 alone). Other groups received this number of D2 cells plus 5×10^6^ CD4_con_, CD4_TGF-β_ or CD4_TGF-β+atRA_. The IgG levels and anti-DNA concentration in mice sera four weeks after cells transfer were determined by ELISA, and proteinuria levels eight weeks after cell transfer were measured using Albustix reagent strips (Bayer, Elkart, IN). Values are Mean ± SEM of 6 mice combined with two separated experiments. (E) Similar experiments were conducted as in panel D. Spleen cells from specific groups of mice 3 weeks after cell transfer were harvested and stimulated with PMA (50 ng/ml), Ionomycin (500 ng/ml) for 5 hrs and BFA (5 µg/ml) for 4 hrs. IFN-γf, IL-4 and IL-17 expression on the CD4+ cells was determined by FACS. Data are Mean ± SEM of three separated experiments. (F) The survival of mice conducted in panel A was monitored. CD4_con_ vs CD4_TGF-β_ (*p*<0.01), CD4_TGF-β_ vs CD4_TGF-β+atRA_ (*p*<0.02) (n = 6, each group).

Previous reports have demonstrated that TGF-β-primed CD4^+^ cells are hypoproliferative themselves yet are able to suppress activation and proliferation of other T cells [9], [11], [16]. As shown in **Figure 2A**, when stimulated with anti-CD3 alone, TGF-β-primed CD4^+^ cells had a low proliferative ability. These cells still produced variable levels of IL-2 (**Figure S1E**), a possible reason for their incomplete anergy status. Nonetheless, the addition of atRA along with TGF-β significantly decreased IL-2 production and almost completely abolished their proliferation, but this could be restored through the addition of exogenous IL-2 (**Figure 2A**). We next examined their suppressive activity in a standard *in vitro* assay. After four days in culture, almost all CD25 expressing cells were also expressing Foxp3, therefore we could use CD4^+^CD25^+^-based magnetic bead isolation to obtain Foxp3+ cells from cells cultured under the following conditions: TGF-β alone, atRA plus TGF-β or control (without TGF-β). These cultured CD4^+^ cells were analyzed in standard suppression assay. As shown in **Figure 2B**, addition of TGF-β-primed CD4^+^CD25^+^ cells to CD25-depleted CD8+ T cells at a ratio of 1∶4 significantly suppressed the CD8^+^ T responder cell proliferation *in vitro*. These cells similarly suppressed CD4^+^ T responder cell proliferation as well *in vitro* (not shown). Conversely, addition of CD4^+^CD25^+^ effector cells (without TGF-β-primed, CD4con) failed to suppress responder T cell response. Of note, addition of similar doses of CD4^+^CD25^+^ cells treated with both atRA and TGF-β resulted in a greater suppressive activity against the proliferative response of CD8^+^ T cells (**Figure 2B**) and CD4^+^ T cells (**Figure 2C**) compared to CD4^+^ cells treated with TGF-β alone. We also observed differential suppressive capacities *in vitro* using purified GFP^+^ Tregs sorted from TGF-β-primed or atRA/TGF-β-primed CD4^+^ cells using Foxp3^gfp^ knock-in mice (**Figure S2**), indicating the increased suppressive effects of CD4^+^ iTregs induced by both atRA and TGF-β are not simply due to only enriched proportion of Foxp3^+^ cell population in this setting.

Suppressive activity *in vitro* does not necessarily reflect a therapeutic effect of Tregs in autoimmune diseases setting *in vivo*. To validate and compare the therapeutic effects of both CD4^+^ Treg populations generated as above, we have used a rapid-read lupus model as previously reported [9]. In this lupus model, the transfer of parental DBA/2 splenocytes to DBA/2 x C57BL/6 F1 mice caused polyclonal B cell activation and anti-dsDNA autoantibodies in 1–2 weeks, and proteinuria in 8–12 weeks [9], [17]. These pathologic effects could be significantly decreased by co-transfer of 5×10^6^ TGF-β-primed CD4^+^CD25^+^ cells. Furthermore, the levels of IgG, anti-dsDNA and proteinuria were markedly lower in lupus mice treated with atRA/TGF-β-primed CD4^+^CD25^+^ cells than in lupus mice treated with TGF-β-primed CD4^+^CD25^+^ cells (**Figure 2D**). 3 weeks after cell transfer, LN cells in DBA/2 x C57BL/6 F1 mice resulted in substantial numbers of Th1 cell (**Figure 2E**) but few Th2 and Th17 cells (not shown) compared to F1 mice receiving no cells. Infusion of TGF-β-primed CD4^+^CD25^+^ cells significantly suppressed the Th1 frequency in lupus mice whereas treatment with atRA/TGF-β-primed CD4^+^CD25^+^ cells resulted in the most significant suppression against Th1 cell production in lupus mice (**Figure 2E**). Accordingly, co-injection of TGF-β-primed CD4^+^CD25^+^ cells but not control (CD4con) cells significantly prolonged the survival of lupus mice in a manner consistent with a previous report (**Figure 2F**) [9]. Of note, the protective effect of atRA/TGF-β-primed CD4^+^CD25^+^ cells on lupus survival was even better. While all lupus mice died in 42 weeks following single co-injection of CD4^+^CD25^+^ cells treated with TGF-β, more than 60% of lupus mice still survived at this time point after receiving CD4^+^CD25^+^ cells treated with both atRA and TGF-β. Taken together, these results indicate that addition of atRA to TGF-β strengthens the quantity and quality of induced Tregs and provide a better approach to treatment of autoimmune diseases and other diseases.

### The ability of atRA to promote Foxp3^+^ regulatory T cell differentiation is TGF-β signaling dependent

**Figure 3:**
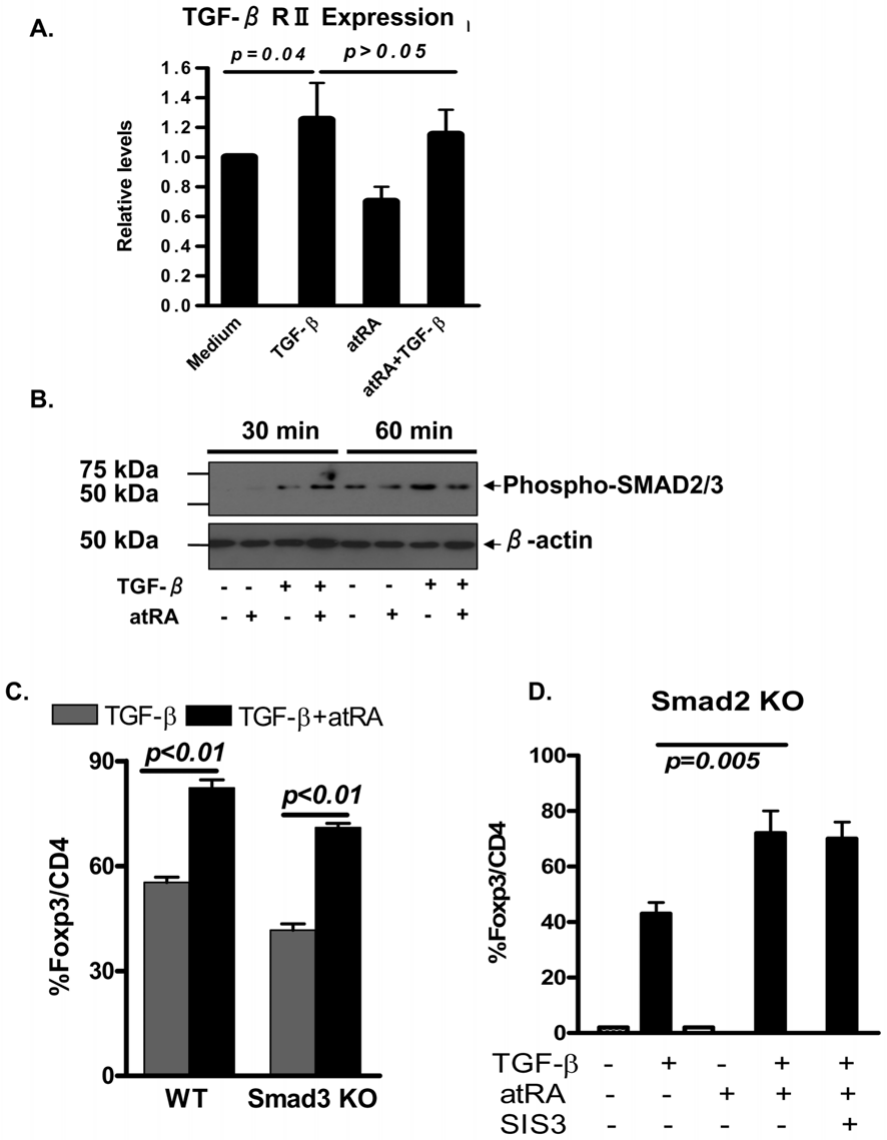
atRA promotes Foxp3 differentiation through Smad independent pathway. (A) Naïve CD4^+^ T cells were activated as in Fig. 1. TβRII expression in different cell groups was analyzed by qPCR. Data are Mean ± SEM of triplicate wells and representative of four separate experiments with similar results. (B) The phosphorylation of Smad2/3 was analyzed by Western blot in different cell groups at 30 min and 60 min. Data are representative of three separate experiments. (C) Naïve CD4^+^ cells isolated from Smad3 KO and wild type mice were TCR stimulated ± atRA ± TGF-β for 4 days and Foxp3 expression was analyzed by FACS. Values are Mean ± SEM of three separate experiments. (D) Naïve CD4^+^ cells were isolated from Smad CKO mice and SIS3 was added to some cultures. Foxp3 analysis was similarly conducted as panel C. Values are Mean ± SEM of three separate experiments.

The inability of atRA alone to induce Foxp3 expression by TCR-stimulated naive CD4^+^CD25^−^ cells (**Figure S1A and C**) indicates that Foxp3 conversion *in vitro* was exclusively dependent on exogenous TGF-β [11], [13]. This finding is exemplified best when using TCR-activated naïve CD4^+^ cells isolated from TGF-β receptor II (TβRII) KO mice. Under these conditions, even a combination of atRA and TGF-β is unable to induce Foxp3 expression (not shown). Using quantitative PCR methods, we now showed that TGF-β increases the TβRII expression by CD4^+^ T cells (**Figure 3A**). atRA alone was unable to increase TβRI (not shown) and TβRII expression, nor did CD4^+^ treated with both atRA and TGF-β (**Figure 3A**), indicating the increased binding ability between TGF-β and TβR in CD4^+^ cells does not appear to contribute to the enhanced effectiveness of atRA to upregulate TGF-β-induced Foxp3 expression.

We next examined whether atRA affects downstream molecules in the TGF-β/TβR pathway, subsequently altering TGF-β's ability to induce Foxp3 expression. The cellular response to TGF-β varies by cell type and by the context of the stimulus. In lymphocytes, TGF-β binds to its cognate receptor complex composed of type I (ALK5) and type II receptors. TβRII is required to activate TβRI in the ligand–receptor complex, and activated TβRI Ser/Thr kinases mainly phosphorylate downstream specific Smad2 and Smad3. We first observed that while TGF-β induces Smad2/3 activation by CD4^+^ cells, the addition of atRA (**Figure 3B**) slightly increased the level of Smad2/3 phosphorylation 30 min after treatment, with this effect disappearing in 60 min after treatment, suggesting that Smad2/3 early activation probably plays a role in the enhancement of atRA to induce Foxp3^+^ cell differentiation that is similar with other reports [18], [19].

Nevertheless, recent studies have demonstrated that Smad2 or Smad3 plays a redundant role in the differentiation of TGF-β-induced Foxp3^+^ cells [11], [20], we therefore further studied the role of atRA in the promotion of iTreg development using Smad2 or Smad3 KO mice. As shown in **Figure 3C**, addition of atRA significantly increased the proportion of Foxp3^+^ cells by WT TGF-β-primed CD4^+^ cells, this phenomenon can be similarly observed in CD4^+^ cells isolated from Smad3 KO mice although the expression levels and intensities of Foxp3 were somewhat lower compared to WT cells. Because conventional Smad2 KO mice are early embryonic lethal, we generated lymphocyte-targeted Smad2 conditional knock-out (CKO) mice by crossbreeding floxed Smad2 and hCD2-Cre mice [11]. As with the Smad3 KO mice, addition of atRA to the TGF-β treated-CD4^+^ Smad2 CKO cultures still resulted in upregulated Foxp3 induction. Moreover, addition of SIS3, a specific Smad3 inhibitor [11], [21], did not alter the levels of Foxp3 expression in Smad2 CKO CD4^+^ cells (**Figure 3D**), excluding the possibility that Smad2 and Smad3 compensate for each other in the promotion of Foxp3^+^ cell differentiation. These results indicate that atRA promotes TGF-β-induced Foxp3^+^ cell differentiation via Smad2- or 3-independent signaling pathways.

**Figure 4:**
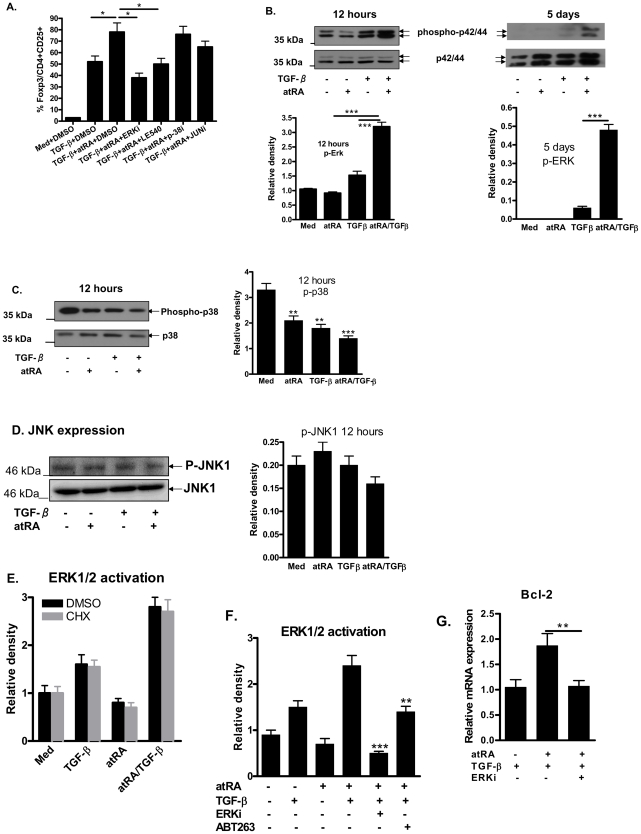
atRA promotes Foxp3 expression through ERK activation and maintenance. (A) Naïve CD4 T cells were stimulated as in Fig. 1. The different MAPKs inhibitors, DMSO or LE540 were added to some cultures. Values indicate Foxp3 expression and are Mean ± SEM of four separate experiments. (B–D) These cells were stimulated as in panel A for 12 and 120 hrs and ERK, p38 and JNK1 activation was examined by Western blot. The data shown are representative of three independent experiments. The relative density of activated ERK, p-38 and JNK1 to total ERK, p-38 and JNK1 was quantified by Quantity One software and values are Mean ± SEM of three separate experiments. (E) Naïve CD4^+^ cells were stimulated as in panel B for 6–12 hrs. In some cultures, cycloheximide (20 µg/ml, Sigma, C1988) or control DMSO were added to cultures for 6 and 12 hrs. Values are Mean ± SEM of relative density of ERK1/2 activation for three separate experiments with 6 hrs stimulation. (F) Experiments were similarly conducted as in panel E and ERKi and ABT263 (Bcl-2 inhibitor, 5 µM, Selleck) were added to cultures for 12 hrs (F) to 5 days (not shown) and ERK1/2 activation was similarly determined as panel E. Values are Mean ± SEM of three separate experiments. *p<0.05, **P<0.01, ***p<0.001 (student *t* test).

In addition to Smad signaling, MAPKs including ERK, JNK, and p38 constitute major non-Smad signaling pathways that play a supplemental role in mediating the intracellular responses to TGF-β. As ERK and JNK mainly contribute to iTreg differentiation [11], we examined whether these non-Smad pathway molecules are involved in iTreg promotion by atRA. In a protocol as similarly described for **Figure S1**, we added different MAPK inhibitors (or DMSO control) to cultures of CD4^+^ T cells incubated with TGF-β. As shown in **Figure 4A**, addition of ERK inhibitors (ERKi) not only completely abolished the increased Foxp3 expression initiated by atRA, but decreased Foxp3 expression levels below that induced by TGF-β itself. We have revealed that this ERKi did not significantly suppress CD25 expression, T cell activation, as well as cell viability (**Figure S3**), excluding the possibility that ERKi suppresses Foxp3 induction because of its global restraint of TCR-stimulation. Addition of JNKi slightly but not significantly abolished the atRA-regulated Foxp3 increase. However, the addition of p38i had no effect on Foxp3 expression. LE540, an RAR antagonist [22], completely abolished the atRA-regulated Foxp3 increase, providing an ideal positive experimental control and further suggests that an atRA/RAR signal is needed for the atRA-regulated Foxp3 increase [15].

Since different subsets of MAPKs may have distinct roles in atRA-mediated promotion of iTreg differentiation, we next asked whether addition of atRA can alter the expression of activated MAPKs in TCR/TGF-β-primed CD4^+^ cells. As shown in **Figure 4B and C**, naive CD4^+^CD25^−^ cells appeared to express activated P42/44 ERK and p38 by 12 hours after stimulating with anti-CD3/CD28 and IL-2 (med) by Western blotting with phosphospecific antibodies. In agreement with a previous report [11], addition of TGF-β alone significantly increased the activation of ERK but suppressed p38 phosphorylation. Addition of atRA alone to the cultures significantly decreased ERK and p38 activation. Notably, addition of both atRA and TGF-β significantly increased P42/44 ERK early activation and late persistence (even after 5-day culture) (**Figure 4B**) although this combination significantly decreased p38 activation (**Figure 4C**) and had less influence on JNK1 activation (**Figure 4D**). Adding a protein synthesis inhibitor, cycloheximide (CHX), to the cultures for 6 hours (**Figure 4E**) and 12 hours (not shown) did not significantly change ERK activation, suggesting that atRA mainly affects ERK activation rather than ERK synthesis (**Figure 4E**). To explain how atRA sustains the ERK activation (up to five days), we have demonstrated that addition of Bcl-2 inhibitor can suppress the ERK early activation (**Figure 4F**) and late maintenance (not shown), suggesting that atRA enhances the cell survival of ERK activated CD4+ cells. Interestingly, addition of ERK inhibitor also suppressed the Bcl-2 mRNA up-regulation on CD4+ cells treated by atRA (**Figure 4G**), implicating the interaction of atRA, ERK and Bcl-2 promotes the development and stability of Foxp3+ Treg cells. Previous studies have reported that atRA inhibits ERK activation in some cell types and activates ERK in others [23], [24]. These results demonstrate that the effect of atRA on the promotion of TGF-β-induced iTreg differentiation appear to be mainly through ERK activation and p38 inactivation. ERK and Bcl-2 interaction may promote the effect of atRA on iTreg promotion and maintenance.

### atRA promotes iTreg development and maintenance through epigenetic modulation of histones rather than through DNA methylation of the Foxp3 gene locus

**Figure 5:**
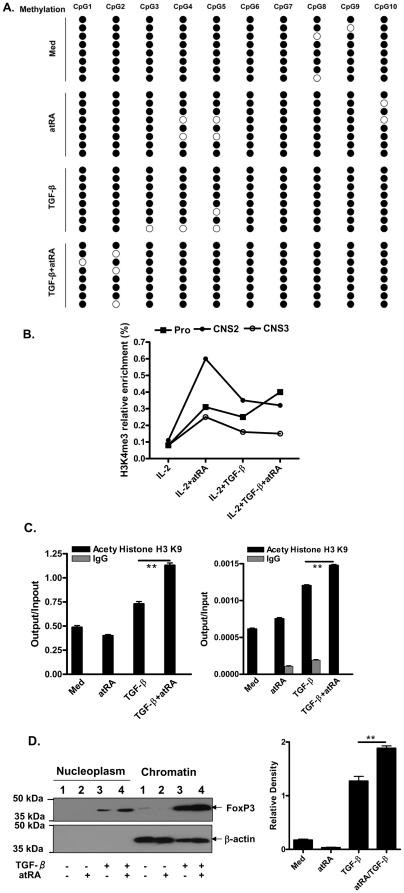
atRA and TGF-β affect histone modification not DNA methylation in the Foxp3 gene locus, and also increase the DNA binding ability of Foxp3 protein. (A) Naive CD4+ cells were stimulated as Fig. 1. The DNA methylation status in CNS3 of the Foxp3 gene locus was determined by bisulfite sequencing analysis. Each line represents one DNA strand; open circle, unmethylated CpGs; filled circle, methylated CpGs. (B) Naïve CD4+ cells were stimulated as in Panel A. ChIP-qPCR detection of H3K4me3 enrichment at promoter, CNS2 and CNS3 of Foxp3. Data are representative of two similar experiments. (C) A ChIP-qPCR analysis was performed to determine histone acetylation in the Foxp3 promoter. The β-actin gene was included as an internal control. Antibodies for N-terminal histone H3 and ser-5-phopho-RNA pol II were used for immunoprecipitation. The DNA fragments bound to the proteins were determined by qPCR analysis. Results showed are Mean ± SEM of four separate experiments. **p<0.01 (student *t* test). (D) Cellular compartments were fractionated into cytoplasmic, nuclear, and chromatin fractions. Equal amounts of proteins were separated by 8% SDS/PAGE, and then transferred to nitrocellulose membrane. Chromatin fractions were immunoblotted successively with antibodies against Foxp3 and β-actin. Relative density of Foxp3 expression was quantified with Quantity One software and values are Mean ± SEM of three separate experiments. **p<0.01 (student *t* test).

Given the critical role of atRA in iTreg promotion and maintenance, we sought to explore possible mechanisms for regulation at epigenetic levels. Others have reported that DNA methylation in Foxp3 gene promoter and CpG sites in the +4,201 to +4,500 intronic CpG island in conserved non-coding DNA sequence 3 (CNS3) at the Foxp3 gene locus affects Foxp3 expression and maintenance by Tregs [25], [26]. We analyzed the DNA methylation in CpG islands in CNS3 of the Foxp3 locus using a previously described method [27]. Potential CpG methylation sites in CNS3 are outlined in **Figure 5A**. Naïve CD4^+^CD25^−^ cells were stimulated with TCR + IL-2 ± atRA and/or TGF-β for 4 days. Of the eight independent clones we noticed that only 1–3 demethylation sites scattered in these ten CpG sites, which may reflect a fluctuation. Each of the different treatments caused a slight shift in the methylation patterns, but overall they were not significantly different. Therefore, atRA-promoted iTreg development and stability does not appear to be related to the alteration of methylation status of CpG sites in CNS3 in the Foxp3 locus.

In addition to DNA CpG site methylation in Foxp3 locus, we also asked whether atRA affects post-translational modification of histones. As the transcribed regions of active genes are usually associated with modification at histone H3K4 [28], we therefore first examined methylation at this histone in the promoter as well as in the CNS elements at the Foxp3 locus. When CD4+ T cells were cultured with atRA or TGF-β, chromatin immunoprecipitations (ChIPs) revealed a significant enrichment for H3K4me3, one of characteristic epigenetic marks of actively transcribed genes for chromatin. The peak accumulation of H3K4me3 was observed on the CNS2 in CD4+ cells treated with atRA alone and on the promoter in the CD4+ cells treated with both atRA and TGF-β (**Figure 5B**), suggesting that while TGF-β promotes histone H3K4me4 methylation level at promoter region of Foxp3 and facilitates iTreg generation, atRA can dramatically enhance histone methylation level at CNS2 of Foxp3 and at least benefit iTreg maintenance. Previous work had documented that CNS3 facilitates Foxp3 induction whereas CNS2 favors the Foxp3 maintenance [29]. We examined histone H3k4me1, H3K4me2 and H3K9 methylation levels in the promoter and CNS1-3 region of Foxp3 locus in CD4+ cells and their enrichments did not correlate with atRA treatment in CD4+ cells (not shown).

In addition to methylation, lysine acetylation is well known for the epigenetic regulation of gene transcription in immune system [28]. Previous work has demonstrated that atRA increases histone acetylation of the Foxp3 promoter regions in natural Tregs [12]. To address whether atRA also affects histone acetylation of the Foxp3 promoter regions in iTregs, we measured the histone acetylation in the proximal 5′ promoter using chromatin immuno-precipitation (ChIP) in conjugation with qPCR. We examined the H3 N-terminal acetylation around the transcription start site (TSS) of Foxp3 gene. As shown in **Figure 5C**, TGF-β alone slightly increased the H3 acetylation in TSS, while atRA alone had no impact. However, atRA in concert with TGF-β significantly boosted the acetylation in the TSS. Phosphorylation at ser-5 and ser-7 of RNA polymerase II is a marker for active transcription [30]. We found that TGF-β but not atRA increased recruitment of phopshorylated RNA pol II in the TSS. Additionally, atRA marginally stimulated additional recruitment of RNA pol II in the promoter. Together, these results suggest that atRA promotes Foxp3 induction and maintenance through modulating epigenetic settings either in a locus-specific manner or globally on chromatin.

We further evaulated the functional outcome of epigenetic modulation regulated by atRA at the Foxp3 locus. To address this, we examined the extent to which atRA stimulation can modify the level of Foxp3 that is bound to chromatin. As shown in **Figure 5D**, we noted significantly greater amounts of Foxp3 in the nuclear fraction (nucleoplasm) 3 days after TCR stimulation with atRA plus TGF-β. Chromatin-associated Foxp3 was found to significantly increase over time in cultures containing both atRA and TGF-β relative to either alone (**Figure 5D**). These results indicate that atRA plus TGF-β stimulation increases the amount of Foxp3 associated with chromatin in CD4^+^ T cells. Increased levels of Foxp3 in the chromatin fraction may facilitate its functional activity during an immune response.

## Discussion

Emerging evidence indicates that atRA promotes the differentiation of TGF-β-induced Foxp3^+^ cells [6]. We have confirmed this finding and also demonstrated that CD4^+^ cells induced by a combination of atRA and TGF-β displayed superior suppressive function *in vitro* and adoptive transfer of these cells resulted in much better suppressive effects on lupus disease development in animal model compared to CD4^+^ cells treated with TGF-β alone, implicating that a combination of atRA and TGF-β provides an ideal protocol for the preparation of iTreg population and their use in the clinical cell therapy in treating autoimmune disease and organ transplantation settings.

The addition of atRA not only increased the differentiation of Foxp3^+^ cells, but also maintained Foxp3 expression by TGF-β-primed CD4^+^ cells. This finding could be explained by increased induction and/or decreased apoptosis. It has been well documented that activated lymphocytes are prone to activation-induced cell apoptosis. Although TGF-β prevents activated lymphocytes from apoptosis, our data demonstrate that a combination of atRA and TGF-β markedly increased the expression of anti-apoptotic protein such as Bcl-2 in CD4^+^ cells, and that these increases are accompanied with decreased numbers of Annexin-V^+^GFP^+^ (apoptotic Foxp3^+^) cells, an indication that these cells from apoptosis, eventually leading to the maintenance of Foxp3 expression. atRA-mediated upregulation of Bcl-2 seems to be dependent upon the RAR- rather than RXR-signal pathway, and this parallels with the role of atRA/RAR signal pathway in the Foxp3 induction [15]. Another possibility is that atRA can expand the Foxp3^+^ cells that had been induced by TGF-β. In addition to the direct effect of atRA on promoting iTreg differentiation, atRA also suppresses the expansion of T effector cells and Th17 cell differentiation induced by a combination of IL-6 and TGF-β [6], [8]. Cytokines produced by effector T cells will diminish the Treg phenotypes and increase the Treg plasticity [19], although others have reported that atRA can interfere directly with decrease in Treg conversion caused by costimulation and that it can enhance Treg conversion from naive T cells independently of secreted cytokines [31]. Recently, the role of atRA in maintenance of expanded nTregs has also been documented [19].

The addition of atRA to TGF-β endows CD4^+^ cells with an almost complete anergic status (**Figure 2A**). This effect could be explained by their decreased IL-2 production and/or an increase in IL-10 production. Our data revealed that CD4^+^ cells treated with TGF-β plus atRA exhibited a lower IL-2 production, a finding which might have contributed to their anergic phenotype. Although IL-10 production by CD4^+^ cells treated with atRA and TGF-β was not markedly increased, its involvement in the role of atRA in iTreg cell development is still unclear. Another group recently reported that the addition of atRA to TGF-β suppresses the production of IL-10 [32]. Our previous work has demonstrated that TGF-β induces iTreg development through IL-10-independent pathway [33].

One of the interesting findings in the current study is that atRA/TGF-β-induced CD4+ regulatory T cells exhibit an enhanced suppressive T cell response *in vitro* and ameliorated lupus syndromes in a chronic GVHD animal model. These findings can not simply be explained by enhanced Foxp3 expression on CD4^+^ cells treated with both atRA and TGF-β compared with TGF-β alone since use of purified individual Foxp3^+^ cells from both groups of cells still displays the similarly functional differences. The remarkable suppressive function of atRA/TGF-β-induced CD4^+^ regulatory T cells in lupus mice may be related to the alteration of the phenotypes, stability and functionality of these cells *in vivo*. Our findings have demonstrated that iTregs induced with atRA/ TGF-β survived longer and mainained higher Foxp3 expression compared to iTregs induced with TGF-β alone *in vivo*.

We have been focusing on an investigation of the molecular mechanism(s) by which atRA promotes the iTreg development and maintenance. An interrupted TβR signaling pathway would abolish the effect of atRA on the increase of Foxp3^+^ cell production, and this suggests that atRA should affect TGF-β signaling rather than TGF-β affects atRA signaling. Previous studies have demonstrated quite clearly that the TGF-β signaling pathway is crucial for the induction of Foxp3^+^ cells [11]. Conversely, the TGF-β-mediated induction is RA receptor α (RAR-α) independent although this receptor is crucial for the effect of atRA on T cell response [12]. In addition, atRA alone without TGF-β is unable to induce Foxp3^+^ iTregs (**Figure S1A and C**).

The two pathways that can regulate TGF-β signaling include the Smad- and non-Smad pathways. Unlike TGF-β, which can upregulate TβRI and II expression by CD4^+^ cells, atRA did not increase TβRI and II expression by CD4^+^ cells (**Figure 3A**), implicating that atRA possibly regulates TGF-β signaling pathway through downstream molecules.

Smad2 and Smad3 are two main targets of activated TβRI and II. Accumulating evidence has revealed that Smad3 is essential for the suppressive effect of TGF-β on IL-2 production, T cell proliferation as well as Th2 type cytokine productions and Th2 type disease in the skin [11]. Although the role of Smad2 or Smad3 in the induction of iTregs is a non-essential one [11], [20], it is unclear whether Smad2/3 is involved in the enhanced effects of atRA on Foxp3^+^ cell differentiation induced by TGF-β. Our data showed that the addition of atRA to TGF-β slightly increased Smad2/3 activation in the 30 min time point but this effect rapidly disappeared by 60 min, implicating that atRA possibly enhances the induction of Foxp3^+^ cells through the early initiation of Smad2/3 activation. One group has also demonstrated that atRA increases the quantity of Foxp3^+^ Tregs by enhancing TGF-β-driven Smad3 signaling while another group recently demonstrated that atRA increases histone acetylation in the Smad3 binding sites and binding ability of activated Smad3 [7], [34]. We found that even when using either Smad3 KO or Smad2 CKO mice, addition of atRA to TGF-β treatment still increased Foxp3^+^ cell induction from TCR-activated CD4^+^ cells although the levels and mean fluorescence intensity (MFI) of Foxp3 were slightly lower than that in WT mice. Another laboratory group has made a similar observation using Smad3 KO mice as well [31]. However, Xu *et al* recently observed that Smad3 is essential for the atRA promotion in Treg differentiation [34]. To explain the difference in the role of Smad3, we would suggest that differences in Treg generation protocols between the two laboratories might contribute to these disparate findings. While Xu *et al* used plate-bound TCR stimulation, our work has used anti-CD3/CD28 coated beads. The exact role of Smad3 in the process of atRA-driven induced Treg development needs to be further explored with different protocols and other Smad3 knock-out strains.

In the current study, we demonstrate that atRA promotes iTregs differentiation through the MAPK pathway. We observed that the addition of ERK inhibitors completely abolished the enhanced effects of atRA on the promotion of TGF-β-induced Foxp3^+^ cells. Using cell activation and proliferation experiments, we showed that ERK inhibitors specifically suppressed the increase of Foxp3^+^ iTreg populations rather than nonspecific suppression of global T cell activation. Consistent with this result, we also observed that the addition of atRA to TGF-β significantly increased ERK activation without enhancing JNK. Conversely, atRA actually suppressed p38 activation. Since p38 activation is associated with T effector cell development and disease pathogenesis [35], atRA may have dual roles in both promoting Treg induction while suppressing T effector cells. The identification of these signaling pathways sheds light on the mechanisms by which atRA promotes the development of the induced Treg subset and will allow for the development of therapeutics that can target specific TGF-β responses in autoimmune and inflammatory diseases.

Our study has also illuminated an intrinsic relationship between Bcl-2 upregulation and ERK activation. ERK activation plays an important role in iTreg induction and promotion of atRA-mediated iTreg differentiation and maintenance. We found that blockade of ERK activation not only suppressed Foxp3 generation, but Bcl-2 upregulation as well. In addition, blockade of Bcl-2 decreases ERK activation, indicating Bcl-2 regulation and interaction of Bcl-2 and ERK may contribute to iTreg maintenance. Future study is needed to determine whether ERK can directly bind Bcl-2 in iTreg cells.

One of the most interesting findings in the current study is that atRA not only promotes iTreg generation and maintenance, but may also alter the binding ability of Foxp3 protein on chromatin. In mammals, epigenetic regulation is mediated by changes in chromatin structure, resulting from either histone modification or DNA methylation [28]. Moreover, we observed that addition of atRA did not alter DNA methylation status in CNS3 sites of the Foxp3 locus in TGF-β-primed CD4^+^ cells. Although others have claimed that incomplete CpG demethylation in Tregs leads to the loss of Foxp3 expression and suppressive activity [25], the addition of atRA enhances and sustains Foxp3 expression in iTregs, and these cells display an exceptionally strong suppressive activity (**Figure 2B–F**), indicating that CpG DNA demethylation of the Foxp3 locus may not be a crucial factor in Treg stability and functionality.

Histone modification represents another possible mechanism in the regulation of gene expression and chromatin structure [36]. For example, histones can be methylated on lysine and arginine (R) residues, acetylated on lysine (K) residues, phosphorylated on serine and threonine (S/T) residues or ubiquitinated/SUMOylated on lysine residues [37]. Histone methylation is more complex and can be associated with either gene activation or gene repression depending on the methylated residue and the degree of methylation, since lysine residues can be mono-, di- or tri-methylated (me1, me2 or me3). In general, the H3K4me3 level indicates gene activation while the H3k27me3 level indicates a repressed gene activity [28]. Our study revealed that atRA or TGF-β treatment can significantly increase the methylation in histone H3K4 in the Foxp3 gene promoter and both atRA and TGF-β treatment upregulated methylation of H3K4 in CNS2 at the Foxp3 locus, indicating that atRA and TGF-β have a synergistic role in the maintenance of Foxp3 but a separate role in Foxp3 induction. Although in an *in vitro* culture, we failed to observe that atRA alone induced Foxp3 expression, others have reported that atRA treatment induced Treg cell-dependent immune tolerance by suppressing both CD4(+) and CD8(+) Teff cells while promoting Treg cell induction and expansion *in vivo* [38]. Future study is needed to determine if atRA also affects the level of histone H3K27me3 modification at the CNS1-3 sites of the Foxp3 locus. Moreover, whether atRA can affect Foxp3 protein acetylation needs to be further addressed since acetylation-mediated Foxp3 protein displayed markedly increased stabilization and functionality [39].

An initial step in the relaxation of chromatin structure is the enzymatic addition of acetyl groups to the positively charged tails of histone H3 and H4 by histone acetyltransferases. Histone acetylation often accompanies gene transcription [40], which is required for the appropriate tissue-specific and context-dependent induction of many genes, and is opposed by the activity of histone deacetylases [41]. We hypothesized that the histone acetylation status of the Foxp3 promoter is responsible for the enhanced effects of atRA on iTreg promotion. Using a ChIP assay, we observed that TGF-β alone can indeed promote Foxp3 acetylation, a finding that is agreement with a previous report [42]. Of note, the histone (H3) acetylation status in iTregs induced by TGF-β and atRA was significantly increased, suggesting that histone/protein deacetylases (HDACs) regulate chromatin remodeling and Foxp3 gene expression and function. It is possible that atRA also affects the histone acetylation in the enhancer region of the Foxp3 gene [34]. Although portions of the total pool of Foxp3 can exist in diverse nuclear sites such as within the nucleoplasm, active and acetylated Foxp3 is preferentially, but not exclusively, bound to chromatin. In fact, histone deactylase inhibitors affect the chromatin binding pattern of Foxp3 [43]. These data suggest that the effect of atRA on Foxp3 mediated regulation of its target gene may be differential and site-dependent, which could explain the differential effect of atRA treatment on the differential induction and function of Foxp3+ Treg cells (**Figure 2**).

In summary, we found that atRA markedly promotes the phenotypic and functional development and maintenance of TGF-β-induced iTregs. Adoptive transfer of these cells to lupus mice highlights their efficient suppressive activity in controlling disease development. We explored the mechanisms involved in the iTreg promotion by atRA, finding that atRA synergizes with TGF-β signaling to induce iTregs mainly via ERK1/2 pathways. Moreover, we observed that atRA significantly increases histone modification including methylation and acetylation but does not affect the DNA demethylation status of CNS3 in the Foxp3 gene locus. We conclude that alterations in the induction and maintenance of Foxp3 gene expression and the conformational changes which promotes its binding on chromatin might both account for increased suppressive activity and stability of iTregs.

## Materials and Methods

### Mice

C57BL/6 mice were purchased from The Jackson Laboratory. *Smad2* ^fx/fx^ and *Smad3* KO mice were provided by Dr. Xiao-Fan Wang at Duke University and Dr. Michael Weinstein at Ohio State University. hCD2-Cre mice were provided by Dr. Dimitris Kioussis at National Institute for Medical Research. *Foxp3 GFP* knock-in mice were a gift from Dr. Talil Chatilla (UCLA). Lymphocyte-specific *Smad2* conditional knockout (CKO) mice were generated by crossing *Smad2* ^fx/fx^ with *Smad2* ^fx/fx^/hCD2-Cre mice. Mice with genotype of *Smad2* ^fx/fx^ were used as a normal control. All animals were treated according to National Institutes of Health guidelines for the use of experimental animal with the approval of the University of Southern California Committee for the Use and Care of Animals (IACUC #11481).

### Cell differentiation and functional assay

Naïve splenic CD4^+^CD25^−^CD44^low^ cells were stimulated with anti-CD3/CD28 beads (Invitrogen, Carlsbad, CA) at a bead/T cell ratio of 1∶5 + IL-2 (20 U/ml, R&D system) ± TGF-β (2 ng/ml, R&D system) for the generation of Foxp3^+^ Tregs for the numbers of days indicated in the different figure legends. 3 µM SIS3 (Smad3 inhibitor, Calbiochem) was added to cultures one hour before TCR stimulation. An equivalent volume of DMSO was added to cultures as a control. To assess suppressive activities, CD4^+^CD25^+^ cells were isolated using MACS beads from CD4^+^ conditioned cells after 4-day culture. T cells labeled with CFSE (Invitrogen, Carlsbad, CA) were stimulated with soluble anti-CD3 (0.025 µg/ml) with irradiated non-T cells as APC (1∶1). The CD4^+^CD25^+^ cell population generated from different groups of mice were added at a ratio of 1∶4 and suppression of cycling CFSE-labeled T cells was assessed as described previously [44]. In other experiments, a [^3^H] thymidine incorporation assay was also used to evaluate the suppressive activity of iTregs [33]. AIM-V serum-free medium (Invitrogen Life Technologies) supplemented with 100 U/ml penicillin, 100 µg/ml streptomycin, and 10 mM HEPES (all obtained from Invitrogen Life Technologies) was used for the generation of CD4^+^ iTreg or control cells. RPMI 1640 medium supplemented as above with the addition of 10% heat-inactivated FCS (HyClone Laboratories) was used for all other cultures.

### Flow cytometry

Anti-TGF-β RII, CD4, CD25, CCR-9,α_4_β_7_,CTLA-4, CD28, CD103, CD126, CD127, CD130 and Foxp3 fix/perm buffer set were purchased from Biolegend (San Diego, CA). Anti-TGF-β was a gift from eBioscience. For intracellular/intranuclear staining, cells were first stained with surface antibodies, then were fixed/permeabilized in cytofix/permeabization solution (Biolegend) and stained with anti-CTLA-4 or anti-Foxp3.

### Real-Time PCR

Total RNA was extracted with the RNeasy mini kit (Qiagen, Valencia, CA). cDNA was generated using a Omniscript RT kit (Qiagen, Valencia, CA). Foxp3 mRNA expression was quantified with ABsolute SYBR Green ROX mix (Thermo, Waltham, MA). The samples were run in triplicate and the relative expression of Foxp3 was determined by normalizing the expression of each target to hypoxanthine guanine phosphoribosyl transferase (HPRT). Primer sequences were as follows: HPRT 5′-TGA AGA GCT ACT GTA ATG ATC AGT CAA C-3′ and 5′-AGC AAG CTT GCA ACC TTA ACC A-3′; Bcl-2, 5′-CCT GGC TGT CTC TGA AGA CC-3′ and 5′-CTC ACT TGT GGC CCA GGT AT-3′. TβRII, 5′-GGC TCT GGT ACT CTG GGA AA-3′ and 5′-AAT GGG GGC TCG TAA TCC T-3′.

### Western blot analysis

Western blot was performed as previously described [44]. Biefly, cells were lysed in buffer containing 25 mM Tris-HCl, 1% deoxycholate, 0.35 M NaCl, phosphatase inhibitor solution (Cayman Chemical), and 1% Triton X-100 (Fischer Scientific). Protein quantity was assayed by bicinchoninic acid (Pierce, Chemical Co.) and 20 µg of protein was loaded per well on a 15% Tris-HCl gel (Bio-Rad). The contents of the gel were transferred in a Trans-Blot semi-dry transfer cell (Bio-Rad) onto nitrocellulose membranes (Amersham Biosciences). The membranes were incubated with different Abs. Ab-bound proteins were detected using an ECL Western blotting analysis system (Amersham Biosciences), and the membranes were exposed to Kodak Biomax XL x-ray film.

### Nuclear and DNA preparation

Nuclear extracts were prepared according to the methods of Li *et al* with some modification [45]. Genomic DNA was prepared using the Qiagen Blood & Cell Culture DNA Kit or the Qiagen DNeasy Blood & Tissue Kit when working with smaller cell numbers. DNA concentration was determined with the NanoDrop spectrophotometer and quality was assessed by agarose gel electrophoresis.

### Cell lysis, immunoprecipitation, and immunoblotting

Cell lysates were obtained by cell lysis in RIPA buffer (50 mM Tris•HCl, pH 7.4, 0.5% Nonidet P-40, 0.25% Na-deoxycholate, 150 mM NaCl, 1 mM EDTA, with 1 mM PMSF, 1 µg/ml each of Aprotinin, leupeptin and pepstatin, 1 mM Na_3_VO_4_, and 1 mM NaF), followed by immunoprecipitation with the indicated antibodies, SDS/PAGE, and analyzed by Western blotting with standard procedures. ECL or ECLplus Western blotting detection reagents were used (Amersham Pharmacia Biosciences).

### Foxp3 methylation analysis

Foxp3 methylation analysis was conducted with a method as previously reported [26]. In brief, genomic DNA was sonicated to a mean fragment size of 350–400 bp. Four micrograms of each sample was incubated with 200 µL of Protein A–Sepharose 4 Fast Flow beads (GE Healthcare) coated with 80 µg of purified MBD-Fc protein in 2 mL of Ultrafree-MC centrifugal filter devices (Amicon/Millipore) for 3 h at 4°C in buffer containing 300 mM NaCl. Beads were centrifuged to recover unbound DNA fragments (300 mM fraction) and subsequently washed with buffers containing increasing NaCl concentrations (350, 400, 450, and 1000 mM). All fractions were desalted using the MinElute PCR purification kit (Qiagen). The distribution of CpG methylation densities of individual MCIp fractions was controlled by qPCR using primers covering the imprinted SNRPN and a genomic region lacking CpGs (empty 6.2). Fractions containing unmethylated DNA (300∼400 mM) or methylated DNA (≥450 mM) were pooled before subsequent labeling.

### ChIP (chromatin immuno-precipitation)

ChIP assays were carried out with 5∼10 million cells with or without stimulation by using EZ-ChIP (cat. 17–371, Upstate Biotechnology) according to the manufacturer's instructions. After sonication on ice, the chromatin solution was centrifuged for 10 min at 6,000× g. mIgG (Upstate Biotechnology), anti-acetyl histone H3 (Upstate Biotechnology), or anti-mono-methyl-histone-K4 H3 (Cell signaling), or anti-di or tri-methyl-histone-K4 H3 (Abcam), or anti-Foxp3 (e-Bioscience) were used for immunoprecipitation of the supernatant. ChIP analysis was carried out according to the manufacturer's protocol (Upstate/Millipore, Billerica, Massachusetts, United States). Cells (1∼5×10^6^) were fixed with 1% formaldehyde, and chromatin was fragmented by ultrasound. For all ChIP samples, sheared chromatin was precleared by incubation with ProteinA-agarose/salmon sperm DNA (Upstate/Millipore). Subsequently, chromatin was immunoprecipitated by overnight incubation at 4°C with 4-µg antibodies (rabbit isotype, #2027, Santa Cruz Biotechnology, Santa Cruz, California, United States; anti–acetyl-histone H3, #06–599, Upstate/Millipore; anti–acetyl-histone H4, #06–866, Upstate/Millipore; anti–tri- or di-methyl-K4-histone H3, ab303938, abcam; and anti-mono-methyl-K4-histone H3, #5326, cell signaling.) followed by incubation with Protein A-agarose/salmon sperm DNA for 1 h. Precipitates were defixed and DNA was purified by using the NucleoSpin Extract II kit (Macherey-Nagel, Düren, Germany). The amount of immunoprecipitated DNA was quantified by real-time PCR with LightCycler (Roche Applied Science, Basel, Switzerland) using SYBR Green and the following *Foxp3* primer pair 5′-GAC TCA AGG GGG TCT CA-3′; 5′-TTG GGC TTC ATC GGC AA-3′. For histone methylation level assay at *Foxp3* regions, following primers were used: Promoter forward primer: 5′-CTGAGGTTTGGAGCAGAAGGA-3′, reverse primer: 5′-TCTGAAGCCTGCCATGTGAA -3′; CNS2 forward primer: 5′-GTTGCCGATGAA GCCCAAT -3′; reverse 5′- ATCTGGGCCCTGTTGTCACA-3′; CNS3 forward primer: 5′- AATGAATG AGACACAGAACTATTAAGATGA -3′; reverse primer: 5′-CAGACGGTGCCACCATGAC -3′.

### Statistical analysis

Results were calculated by using GraphPad Prism 4.0 software (GraphPad Spftware, San Diego, CA) are presented as mean ± SEM. Differences in Kaplan-Meier survival curves were analyzed by the log-rank test. Student *t* test was used to assess statistical significance between two groups, and one-way ANOVA and/or non-parametric tests were used to assess statistical significance among multi-groups. Protein quantification in Western Blot was analyzed by Quantity One software (Bio-Rad, Hercules, CA). P value<0.05 is considered as statistically significant difference.

This work was presented in the plenary session of the 2009 annual conference of American College of Rheumatology in Philadelphia.

## Supporting Information

Figure S1 **atRA enhances Foxp3 expression induced by TGF-β in CD4^+^ T cells.** Naïve CD4^+^ T cells isolated from C57BL/6 (A) or Foxp3^gfp^ (B) spleen using magnetic beads were stimulated by anti-CD3/CD28 beads ± TGF-β ± atRA for 4 days. Foxp3 expression was analyzed by flow cytometry. Values are Mean ± SEM (A) and representative (B) of five separate experiments. (C) Foxp3 expression by these cells was analyzed by Western Blot (atRA, 0.2 µM). Results are representative of three separate experiments. (D) Absolute numbers of Foxp3^+^ (GFP^+^) cells were presented in different time points of these cells. Mean ± SEM of four independent experiments is shown. (E) Cell phenotypes were analyzed by two different cell subsets. Data is representative of four separate experiments.
(TIF)

Figure S2 **Purified CD4^+^Foxp3^+^ cells induced by atRA and TGF-β resulted in increased suppressive activity** ***in vitro***. Naïve CD4^+^ T cells isolated from Foxp3^gfp^ spleen using magnetic beads were stimulated by anti-CD3/CD28 beads ± TGF-β ± atRA for 4 days. Foxp3 (GFP) expression by CD4^+^ cells was analyzed and sorted by flow cytometry after cultures. Freshly sorted GFP^+^ (nTregs) and GFP^−^ (control) cells in Foxp3^gfp^ mice were served as positive or negative controls. These cells were added to anti-CD3-stumlated GFP^−^ T responder cells in the presence of APC and their suppressive activity was analyzed by thymidine [H^3^] incorporation assay as previously described [16]. Mean ± SEM of triplicate experimental data in each group is shown. Data is representative of three separate experiments.
(TIF)

Figure S3 **MAPK inhibitors did not affect the cell viability**. Naïve CD4 T cells were stimulated with anti-CD3/CD28 coated beads ± TGF-β ± atRA for 4 days. The different MAPKs inhibitors, DMSO or LE540 were added to some cultures. Total viable cell numbers were counted in each well. Values indicate viable cell counts and are Mean ± SEM of four separate experiments.
(TIF)
