# Supplementary figures and images for: All-Trans Retinoic Acid Promotes TGF-β-Induced Tregs via Histone Modification but Not DNA Demethylation on Foxp3 Gene Locus
Source: PLoS One. 2011 Sep 13;6(9):e24590. doi: 10.1371/journal.pone.0024590 (PMC3172235; doi:10.1371/journal.pone.0024590)

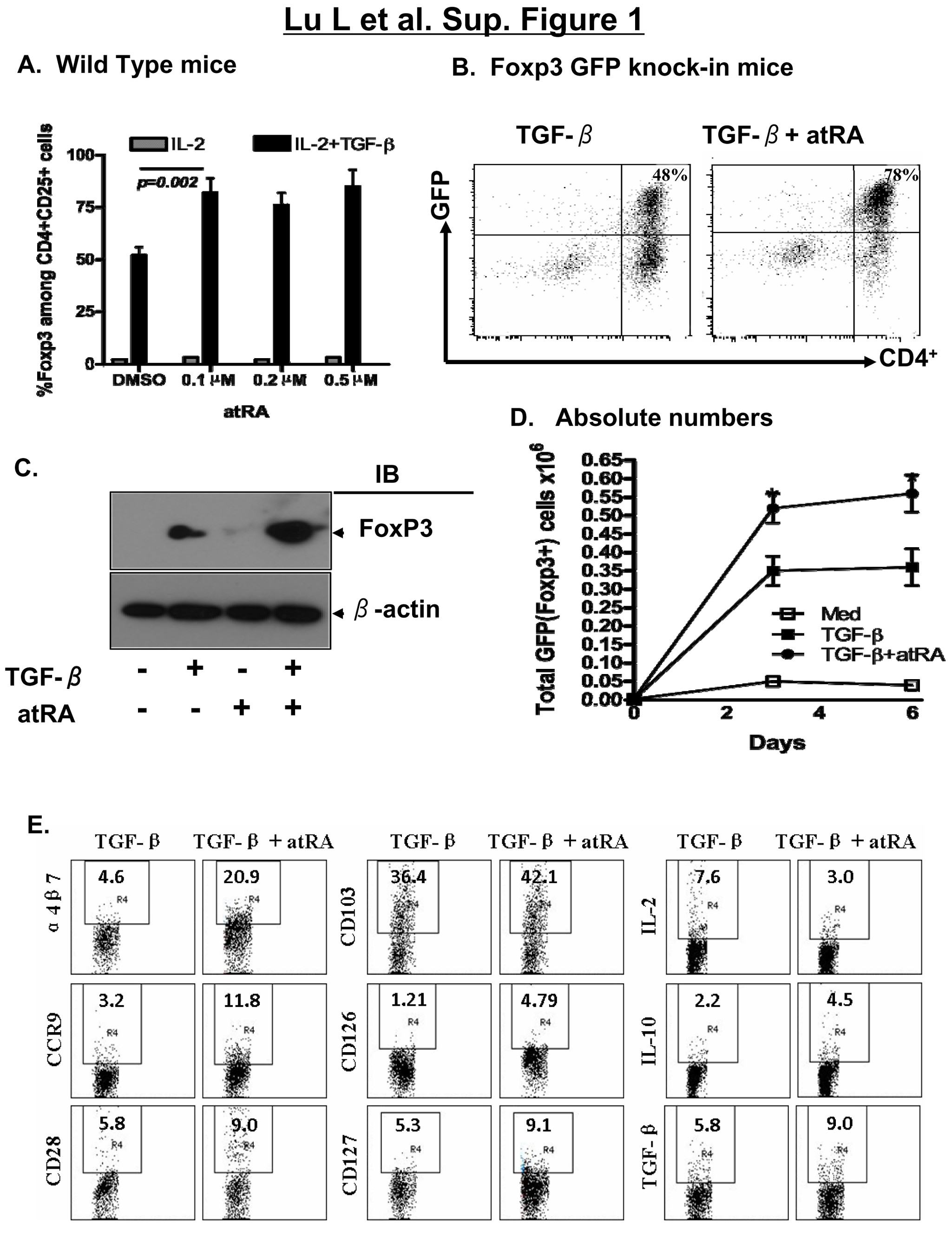

Supplement: Figure S1 — atRA enhances Foxp3 expression induced by TGF-β in CD4+ T cells. Naïve CD4+ T cells isolated from C57BL/6 (A) or Foxp3gfp (B) spleen using magnetic beads were stimulated by anti-CD3/CD28 beads ± TGF-β ± atRA for 4 days. Foxp3 expression was analyzed by flow cytometry. Values are Mean ± SEM (A) and representative (B) of five separate experiments. (C) Foxp3 expression by these cells was analyzed by Western Blot (atRA, 0.2 µM). Results are representative of three separate experiments. (D) Absolute numbers of Foxp3+ (GFP+) cells were presented in different time points of these cells. Mean ± SEM of four independent experiments is shown. (E) Cell phenotypes were analyzed by two different cell subsets. Data is representative of four separate experiments. (TIF) [file pone.0024590.s001.tif]

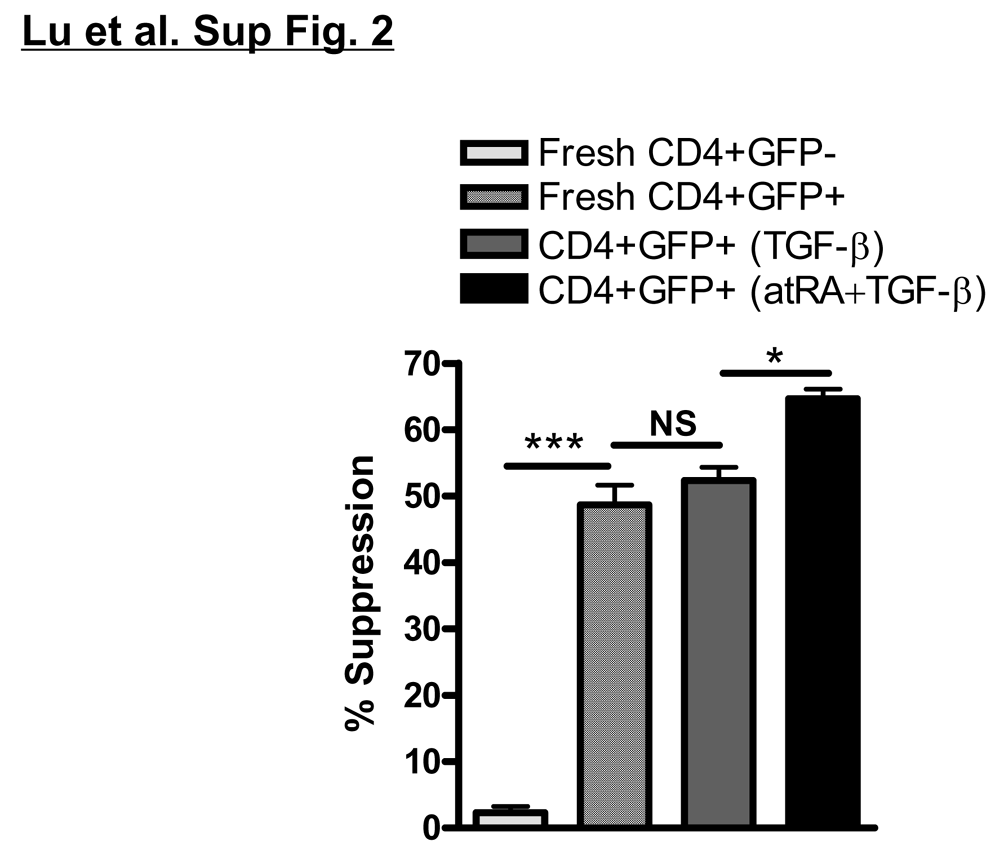

Supplement: Figure S2 — Purified CD4+Foxp3+ cells induced by atRA and TGF-β resulted in increased suppressive activity in vitro. Naïve CD4+ T cells isolated from Foxp3gfp spleen using magnetic beads were stimulated by anti-CD3/CD28 beads ± TGF-β ± atRA for 4 days. Foxp3 (GFP) expression by CD4+ cells was analyzed and sorted by flow cytometry after cultures. Freshly sorted GFP+ (nTregs) and GFP− (control) cells in Foxp3gfp mice were served as positive or negative controls. These cells were added to anti-CD3-stumlated GFP− T responder cells in the presence of APC and their suppressive activity was analyzed by thymidine [H3] incorporation assay as previously described [16]. Mean ± SEM of triplicate experimental data in each group is shown. Data is representative of three separate experiments. (TIF) [file pone.0024590.s002.tif]

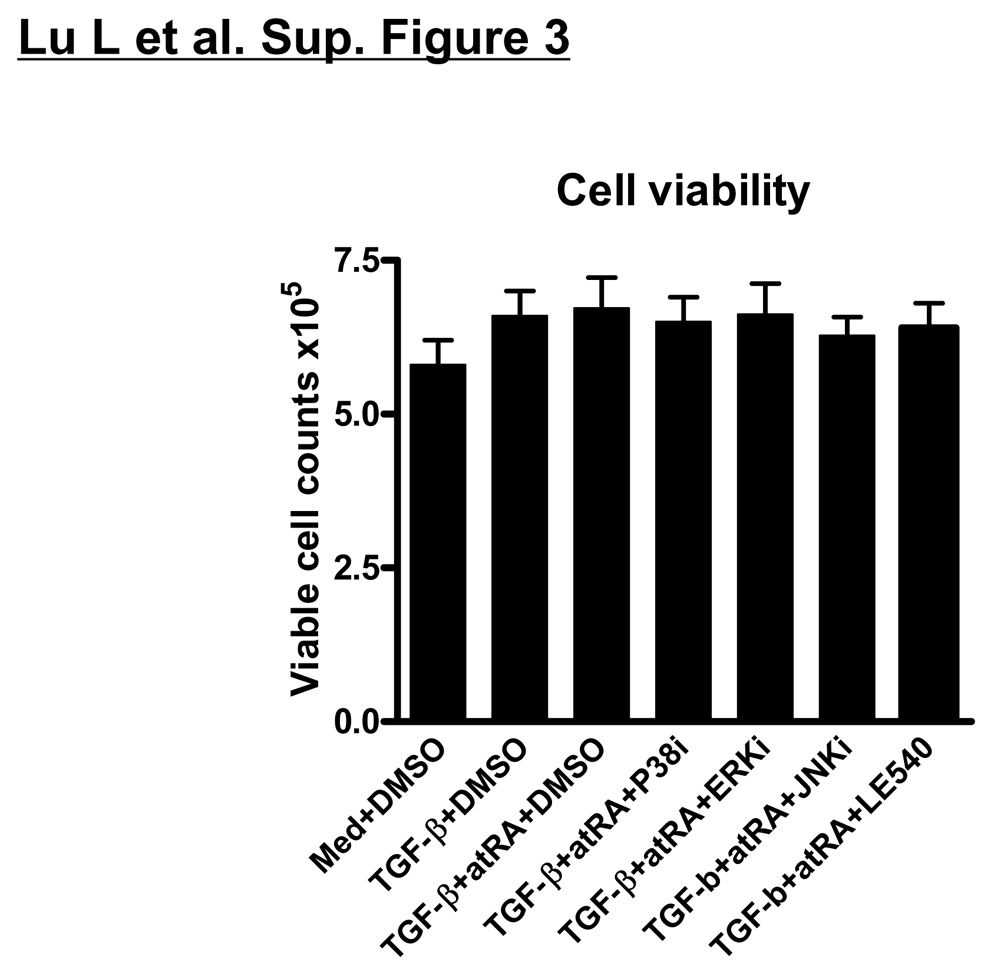

Supplement: Figure S3 — MAPK inhibitors did not affect the cell viability. Naïve CD4 T cells were stimulated with anti-CD3/CD28 coated beads ± TGF-β ± atRA for 4 days. The different MAPKs inhibitors, DMSO or LE540 were added to some cultures. Total viable cell numbers were counted in each well. Values indicate viable cell counts and are Mean ± SEM of four separate experiments. (TIF) [file pone.0024590.s003.tif]
